# Genome-wide association study of neck circumference identifies sex-specific loci independent of generalized adiposity

**DOI:** 10.1038/s41366-021-00817-2

**Published:** 2021-04-27

**Authors:** Yaowu Liu, Xiaoyu Zhang, Jiwon Lee, Diane Smelser, Brian Cade, Han Chen, Hufeng Zhou, H. Lester Kirchner, Xihong Lin, Sutapa Mukherjee, David Hillman, Ching-Ti Liu, Susan Redline, Tamar Sofer

**Affiliations:** 1 Department of Biostatistics, Harvard T.H. Chan School of Public Health, Boston, MA USA; 2 Department of Biostatistics, Boston University School of Public Health, Boston, MA USA; 3 Division of Sleep and Circadian Disorders, Brigham and Women’s Hospital, Boston, MA USA; 4 Department of Molecular and Functional Genomics, Geisinger Clinic, Danville, PA USA; 5 Department of Medicine, Harvard Medical School, Boston, MA USA; 6 Human Genetics Center, Department of Epidemiology, Human Genetics, and Environmental Sciences, School of Public Health, The University of Texas Health Science Center at Houston, Houston, TX USA; 7 Center for Precision Health, School of Biomedical Informatics, The University of Texas Health Science Center at Houston, Houston, TX USA; 8 Department of Population Health Sciences, Geisinger Clinic, Danville, PA USA; 9 Department of Statistics, Harvard University, Cambridge, MA USA; 10 Sleep Health Service, Respiratory and Sleep Services, Southern Adelaide Local Health Network, Adelaide, SA Australia; 11 Adelaide Institute for Sleep Health, Flinders Health and Medical Research Institute, College of Medicine and Public Health, Flinders University, Adelaide, SA Australia; 12 School of Human Sciences, The University of Western Australia, Perth, WA Australia

**Keywords:** Development, Genetics

## Abstract

**Background/objectives:**

Neck circumference, an index of upper airway fat, has been suggested to be an important measure of body-fat distribution with unique associations with health outcomes such as obstructive sleep apnea and metabolic disease. This study aims to study the genetic bases of neck circumference.

**Methods:**

We conducted a multi-ethnic genome-wide association study of neck circumference, adjusted and unadjusted for BMI, in up to 15,090 European Ancestry (EA) and African American (AA) individuals. Because sexually dimorphic associations have been observed for anthropometric traits, we conducted both sex-combined and sex-specific analysis.

**Results:**

We identified rs227724 near the Noggin (*NOG)* gene as a possible quantitative locus for neck circumference in men (*N* = 8831, *P* = 1.74 × 10^−9^) but not in women (*P* = 0.08). The association was replicated in men (*N* = 1554, *P* = 0.045) in an independent dataset. This locus was previously reported to be associated with human height and with self-reported snoring. We also identified rs13087058 on chromosome 3 as a suggestive locus in sex-combined analysis (*N* = 15090, *P* = 2.94 × 10^−7^; replication *P* =0.049). This locus was also associated with electrocardiogram-assessed PR interval and is a cis-expression quantitative locus for the PDZ Domain-containing ring finger 2 (*PDZRN3*) gene. Both *NOG* and *PDZRN3* interact with members of transforming growth factor-beta superfamily signaling proteins.

**Conclusions:**

Our study suggests that neck circumference may have unique genetic basis independent of BMI.

## Introduction

Increased body fat is associated with a range of adverse health conditions, such as metabolic syndrome [1, 2], insulin resistance [3], type 2 diabetes (T2D) [4], cardiovascular diseases [5], and obstructive sleep apnea (OSA) [6]. Body weight is most commonly estimated by calculating the body mass index (BMI). Genome-wide association studies (GWAS) in large cohorts have identified hundreds of BMI-associated loci and improved our understanding of the genetic basis of BMI [7, 8]. However, BMI does not differentiate between fat and lean body mass nor characterize differences in body-fat depots, limiting its ability to characterize body composition or identify individuals with differences in body-fat distribution. Indeed, BMI was developed to characterize weight rather than fat [9]. More direct measures of body-fat distribution obtained through anthropometric measurements include waist circumference, hip circumference, and waist-to-hip ratio. Independent of BMI, large GWAS identified many unique loci for these traits [10], suggesting that there are specific genetic determinants of different body-fat depots.

Sexual dimorphism of adiposity-related traits is well-recognized. Subtle sexual dimorphisms in body-fat distribution emerge in early childhood, and become more apparent during puberty. Central and visceral fat are higher in men, while total, lower body, and subcutaneous fat are greater in women [11–14]. There are large sex differences not only in body-fat depots, but also in adipose tissue metabolism, including adipogenesis, fatty acid metabolism, and gene expression [15], mechanisms that influence propensity for cardiometabolic disorders. Men and women also differ in the rate of accumulation of fat with aging: while visceral and subcutaneous fat both increase with age, a longitudinal analysis of 472 non-Hispanic white adults aged 18–84 years studied twice ~7 years apart found that the rate of change in fat was lower in men than women, although the ratio of visceral to subcutaneous fat remained higher in men compared to women over time [16]. In addition, many anthropometric phenotypes have been shown to have sex-dimorphic genetic associations [14].

Neck circumference (NC) is an index of upper-body subcutaneous fat that correlates with other measures of general and regional adiposity, but appears to be a unique, pathologic fat depot. Specifically, independent of other adiposity indices, NC associates with elevations in blood pressure, triglycerides, low density lipoproteins, and fasting glucose and insulin [17]. Increased NC also is a marker of cardiovascular risk [18] and obesity [19] in children. Similar to other fat depots, NC displays sexual dimorphism. NC is larger in males than females and varies by sex in its association with cardiovascular risk factors [17, 20, 21]. For example, NC was found to associate with diastolic blood pressure in men only; triglycerides and fasting plasma glucose in women only; and insulin and high-density lipoprotein in both men and women [17]. A cross-sectional study also indicated that NC was associated with metabolic syndrome independently of waist circumference or BMI, and the association was stronger in men than women [22]. NC is a strong predictor of OSA, a common disorder associated with obesity and metabolic dysfunction that is more common in men than women. Moreover, increased NC predicts OSA in individuals with and without obesity [23], and therefore is incorporated into OSA screening tools to identify individuals at high risk for the disorder. These findings support the importance of NC as a unique fat depot and the need to better understand determinants of NC in both men and women, which may be facilitated by identifying genetic influences on NC. However, to the best of our knowledge, there are currently no large-scale GWAS for detecting NC-associated loci and the genetic bases for NC largely remain unknown.

Thus, given the previously observed strong evidence of sex-specific associations for other adiposity measures, we performed GWAS of NC in men and women separately, and then performed sex-combined analysis. We conducted a meta-analysis of GWAS involving a total of 15,090 individuals of European ancestry (EA) and African Americans (AA) from seven cohorts. Following the previous GWAS of waist circumference and waist-to-hip ratio, we analyzed NC both with and without adjustment for BMI as a way of inferring associations that may operate independently of BMI.

## Material and methods

### Study subjects

We included seven cohorts in the discovery analyses: the Cardiovascular Health Study (CHS, *n* = 759 EA, 206 AA), the Cleveland Family Study (CFS, *n* = 692 EA, 724 AA), the Framingham Heart Study (FHS, *n* = 6950 EA), the Atherosclerosis Risk in Communities (ARIC, *n* = 1447 EA), the Osteoporotic Fractures in Men Study (MrOS, *n* = 2195 EA), the Western Australian Sleep Health Study (WASHS, *n* = 1582 EA), and the Jackson Heart Study (JHS, *n* = 535 AA). Detailed descriptions of each cohort are provided in the Supplementary Information. We determined that the sample size is adequate for making replicable findings based on other GWAS that used similar sample sizes.

### Phenotypes and covariates

In each cohort, NC was measured in centimeters using a tape measure at the level of the anterior border of the cricoid cartilage or inferior to the laryngeal prominence, perpendicular to the long axis of the neck. The original NCs without adjustment for BMI (NC) and the neck circumference with adjustment for BMI (NCadjBMI) were analyzed separately as two phenotypes. Covariates include age and sex.

### Genotyping and quality control

Genotypes were obtained from dbGaP for ARIC (Affymetrix 6.0; dbGaP phs000035.v1.p1), CHS (Illumina CNV370, ITMAT-Broad-CARe [IBC], and Omni 1M [AA only]; phs000135.v1.p1, phs000077.v1.p1), FHS (Affymetrix 500k mapping array plus Affymetrix 50K supplemental array; phs000006.v7), and JHS (Affymetrix 6.0; phs000104.v1). CFS genotypes were based on Illumina OmniExpress+Exome and IBC arrays (AA and EA), as well as Affymetrix 6.0 and Illumina Exome (AA only). CFS data are available from dbGaP (phs000284.v2.p1). MrOS and WASHS genotyping was performed with the Illumina Omni 1M and 2.5 arrays, respectively. MrOS data are available from dbGaP (phs000373.v1.p1). WASHS data are available by collaboration with WASHS investigators. Individual cohort quality control (QC) measures included exclusions for missingness >5%, Hardy–Weinberg *P* values < 1 × 10^−6^, and duplicate positions. Variant genomic positions were “lifted over” to the Build 37 forward strand using the Strand database and the Haplotype Reference Consortium (HRC) imputation preparation script (https://www.well.ox.ac.uk/~wrayner/tools/) and confirmed using Ensembl variant allele checks and internal QC performed on the Michigan Imputation Server. Study-level data were imputed separately using the HRC v1.1 reference panel [24].

### Statistical analysis

The traits NC and NCadjBMI were analyzed separately in each cohort and population, adjusting for age, sex, and the first three genetics principal components. Since NC and NCadjBMI are approximately normally distributed, we did not perform transformations and the original phenotypes were used in the analysis. We used GEMMA 0.94.1 [25] (coxme [26], an R package, for FHS, function “lmekin”) to fit a linear mixed model with a genetic relatedness matrix (a kinship matrix for FHS) to control for population stratification and relatedness. We then applied inverse variance weighted fixed-effects meta-analysis on the cohort- and ethnic-specific results using METAL [27] with genomic control correction to control for potential type-I error rate inflation due to population stratification. The sex-stratified analyses were conducted in the same manner as the sex-combined analyses, except without sex adjustment. We report significant genetic associations that passed the standard genome-wide significance threshold (*P* < 5.0 × 10^−8^), and “suggestive” associations (*P* < 1.0 × 10^−6^).

### Phenotype association lookups of significant and suggestive loci

To investigate whether our significant and suggestive loci are also associated with other phenotypes, we performed phenotype association lookups of these loci. Specifically, we used the GWAS catalog (https://www.ebi.ac.uk/gwas/), the Cardiovascular Disease Knowledge Portal (http://www.broadcvdi.org/), and the T2D portal (http://www.type2diabetesgenetics.org/) to identify other traits associated with the top loci. We report the additional genetic associations for loci that are associated with at least one trait at the genome-wide significance level (*P* < 5.0 × 10^−8^). In addition, we did lookups for OSA-related traits using the Sleep Disorders Knowledge portal (http://sleepdisordergenetics.org/) and in summary statistics from a GWAS of snoring [28] available from the GWAS Central website [29]. Due to the connection between NC and OSA, we report these lookups for all suggested and significant associations, regardless of their significance level in their association analysis with OSA-related traits.

### Expression quantitative loci lookups and colocalization analysis

We used the expression quantitative loci (eQTLs) databases (i.e., GTEx Portal, https://gtexportal.org/home/) to identify genes that had significant eQTL related to the top and suggestive loci across tissues. We focused on single-nucleotide polymorphisms (SNPs) that reach the genome-wide significance either with NC in our analysis or with other traits in the literature. For the identified genes and tissues that may be related to NC, either through effects on anatomic structures (e.g., muscle, fat, esophagus), or indirectly via production of hormones that affect muscle and fat (e.g., adrenal gland, pancreas), we tested colocalization between the expression association and our NC GWAS association loci using a Bayesian statistical methodology [30], implemented in the R package *coloc*. The default prior probability of colocalization (10^−5^) was used.

### Replication analysis: the Geisinger dataset

We replicated the associations identified in the discovery analysis in an independent dataset of *n* = 3297 European Americans from Geisinger, which is a large integrated health system serving patients across 45 of 67 counties in Pennsylvania. Details on the participants, genotyping, quality control, and statistical analysis of this dataset are provided in the Supplementary Information.

## Results

### Sample description

Key characteristics of each cohort are presented in Table 1, with additional detailed descriptions of the cohorts in the Supplementary Information. Across the seven distinct cohorts, data were available for 15,090 individuals: 6358 women and 8732 men. On average, participants were in middle to late adulthood and had overweight or obesity. In general, the distributions of NC were similar across the cohorts and men had a larger NC than women. Overall, 9.7% of the individuals were of African ancestry and were 90.3% of European ancestry.

**Table 1:** Sample characteristics.

Ethnic group	Cohort	*N*	Age (years)	Percent female	BMI (kg/m^2^)	Neck circumference (cm)
African ancestry	CFS	724	37.7 (19.4)	56.7	31.7 (9.9)	38.0 (5.5)
	CHS	206	75.6 (4.7)	60.4	28.7 (4.8)	37.6 (3.4)
	JHS	535	63.0 (10.8)	63	32.2 (7.2)	39.0 (3.9)
European ancestry	ARIC	1447	62.4 (5.7)	51.6	28.8 (5.1)	38.1 (4.4)
	CFS	692	41.6 (19.4)	52.7	30.3 (8.7)	37.6 (5.3)
	CHS	759	77.8 (4.3)	60.3	27.2 (4.4)	37.3 (3.7)
	FHS	6950	48.4 (13.0)	53	27.3 (5.3)	36.9 (4.3)
	MrOS	2195	76.7 (5.6)	0	27.2 (3.8)	39.4 (2.8)
	WASHS	1582	52.0 (13.7)	40.3	32.2 (8.1)	40.8 (4.7)

The cohorts are: *CHS* Cardiovascular Health Study, *CFS* Cleveland Family Study, *FHS* Framingham Heart Study, *ARIC* Atherosclerosis Risk in Communities, *MrOS* Osteoporotic Fractures in Men Study, *WASHS* Western Australian Sleep Health Study, *JHS* Jackson Heart Study.

### Sex-combined and sex-stratified analyses

**Fig. 1:**
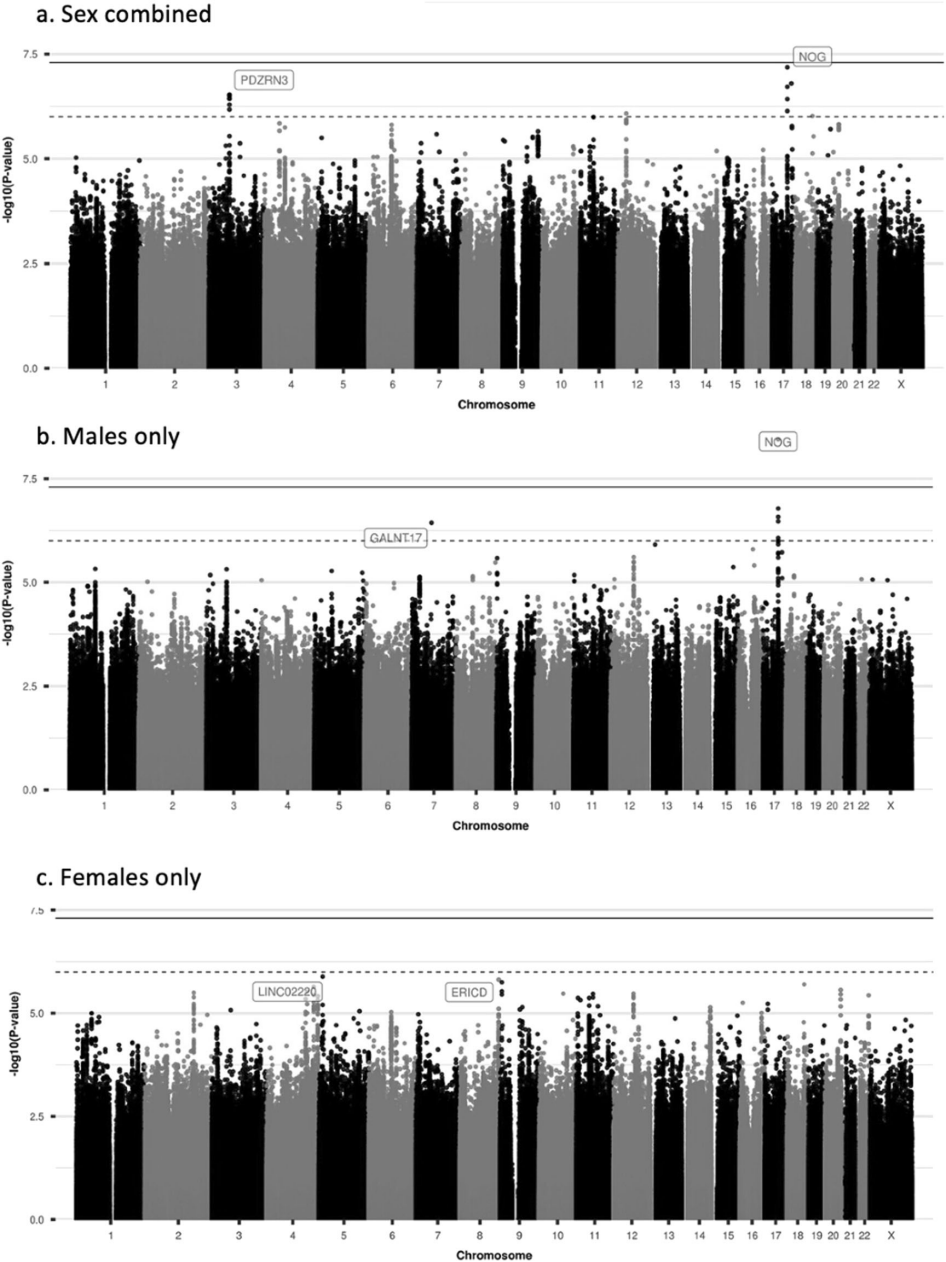
Manhattan plots of sex-combined analysis results for NCadjBMI. The three panels from top to bottom correspond to the results from sex-combined analysis (**a**), males only (**b**), and females only (**c**).

Figure 1 provides Manhattan plots from the the sex-combined and sex-stratified multiethnic meta-analyses for NCadjBMI, highlighting the likely genes of the top association loci. Table 2 presents six genome-wide significant or suggestive SNPs from these GWAS. Genome-wide significant associations were detected only in sex-stratified results. There was one locus significantly associated with NCadjBMI in men on chromosome 17, with a lead SNP rs227724 (*P* = 1.74 × 10^−9^). This SNP is located in an intergenic region between gene Noggin (*NOG)* and gene *C17orf67* and was also reported to be significantly associated with human height [31]. In sex-combined results, five loci showed suggestive significance (*P* < 1.0 × 10^−6^) with NCadjBMI, including one lead SNP rs13087058 that had been previously reported to be significantly associated with the PR interval [32]. No significant associations were observed in women alone.

**Table 2:** Top significant or suggestive associations for NCadjBMI.

				Sex-combined			Men			Women		
SNP	Chr	Gene	Alleles	*N*	Effect	*P*	*N*	Effect	*P*	*N*	Effect	*P*
rs227724	17	NOG	A/T	15090	−0.1367	6.59E − 08	8331	−0.1985	1.74E − 09	6768	−0.0643	7.82E − 02
rs113515681	17	LOC105371884	T/G	13625	−0.4338	1.59E − 07	7430	−0.4828	7.93E − 06	5896	−0.3187	8.02E − 03
rs13087058	3	PDZRN3	T/C	15090	−0.1288	2.94E − 07	8331	−0.139	1.76E − 05	6768	−0.1089	3.05E − 03
rs10771543	12	OVCH1	A/G	15090	−0.1193	8.29E − 07	8331	−0.1152	2.49E − 04	6768	−0.1298	2.10E − 04
rs62101076	18	LOC101927404	A/G	13625	0.4325	9.52E − 07	7430	0.3377	3.62E − 03	5437	0.5491	3.16E − 05
rs139177122	7	RN7SKP75	A/G	14251	−0.4184	1.49E − 06	7430	−0.5769	3.67E − 07	5531	−0.1511	2.54E − 01

Table 1 in the Supplementary Information presents SNPs from ten loci with genome-wide or suggestive significance in the multiethnic combined or sex-stratified meta-analyses for NC (unadjusted for BMI). A lead SNP rs56094641 (*P* = 1.48 × 10^−9^) on chromosome 16 reaches the genome-wide significance in sex-combined analysis. This SNP is an intronic variant in the fat mass and obesity-associated, alpha-ketoglutarate-dependent dioxygenase (*FTO)* gene and was previously identified to be associated with multiple other traits, including BMI and T2D [33].

The lead SNP rs227724 is substantially less significant in analyses unadjusted for BMI compared to BMI-adjusted analyses, and this is observed in both sex-combined and sex-stratified analyses (Supplementary Table 1), which may indicate that it affects NC through pathways independent of BMI. With additional adjustment for height, the association of rs227724 is modestly attenuated in sex-combined analysis for both NCadjBMI and NC (*P* = 1.66 × 10^−6^ for NCadjBMI and *P* = 2.18 × 10^−6^ for NC), suggesting that only a portion of the association is mediated through factors associated with height.

### Associations of top SNPs with other phenotypes

We looked up our top association in the GWAS catalog, the Cardiovascular Disease Knowledge portal, and the T2D portal. Three of the lead SNPs, rs227724, rs13087058 and rs56094641, had genome-wide significant associations with at least one trait. We report their *P* values for other potentially relevant traits in Supplementary Table 2. Specifically, for the two NCadjBMI-associated SNPs, only the association between rs227724 and height, and the association between rs13087058 and PR interval reached the genome-wide significance level. The SNP rs56094641 is associated with BMI as well as several other traits (e.g., T2D).

As NC is a risk factor for OSA, we reported the *P* values of our NC and NCadjBMI-associated top SNPs for OSA-related traits (BMI-adjusted) in Supplementary Tables 3 and 4. Only one association was highly significant: the *NOG* SNP, rs227724, was associated with snoring (BMI-adjusted, *P* = 6.5 × 10^−6^). Associations with OSA-specific phenotypes were mostly not significant. The association with the lowest *P* value was observed for sleep-related hypoxemia (percent sleep time under 90% oxyhemoglobin saturation, sex-combined) with the SNP rs10771543 (*P* = 0.02). This SNP was suggestively associated with NCadjBMI in our sex-combined analysis.

### Expression quantitative trait loci

We examined the association of our top loci with gene expression in specific tissues and cell lines using the GTEx Portal (release 7; https://gtexportal.org/home/). We report the results for SNPs that reach the genome-wide significance either with NC in our analysis or with other traits in the literature. The SNP rs227724 is associated with the expression of the gene *NOG* in multiple tissues (Supplementary Fig. 1, e.g., esophagus-muscularis, *P* = 3.4 × 10^−8^; adipose-subcutaneous, *P* = 4.5 × 10^−3^). The SNP rs13087058, reported to be associated with PR interval, is also associated with PDZ Domain-containing ring finger 2 (*PDZRN3*) gene expression in esophagus and artery-related tissues (Supplementary Fig. 2, e.g., artery-tibial, *P* = 9.3 × 10^−30^). The SNP rs56094641, associated with NC without BMI adjustment, shows a moderate association with the expression of the gene *FTO* only in the muscle-skeletal tissue (*P* = 1.6 × 10^−5^, Supplementary Fig. 3).

### Fine-mapping using colocalization analysis

We focus on the significant genes for the three top SNPs (rs227724, rs13087058, and rs56094641) from eQTL analysis and select several tissues that may relate to the development of NC to conduct colocalization analysis. For each pair of gene and tissue, we report the most likely causal SNP associated with both the trait (NC or NCadjBMI) and the gene’s expression in the tissue. The results are presented in Supplementary Table 5. The highest posterior probability for the evidence of colocalization was only 0.24 for the *PDZRN3* gene in artery-tibial tissue at the locus rs13087058. While we do not observe strong evidence of colocalization, the results also suggest the most likely causal SNPs for the expression of genes in different tissues.

### Assessment of generalizability in independent samples

We tested the associations reported in Table 2 for replication in an independent dataset from Geisinger. Details about the subjects of this replication dataset are provided in Supplementary Table 6. We only conducted replication analysis with BMI-adjusted associations because the top BMI-unadjusted association is in the FTO gene and a known obesity association. Table 3 provides the one-sided *P* values [34] in the replication analysis. Our top association rs227724 was replicated in men (*P* = 0.045), and the suggestive association rs13087058 also showed evidence for replication in sex-combined result (*P* = 0.049). We further meta-analyzed these results. Rs227724 passed the genome-wide threshold in sex-combined results (1.3 × 10^−8^), with a stronger association in male-specific analysis (*P* = 4.2 × 10^−10^), as expected. Rs13087058 also showed stronger results in the meta-analysis but did not reach genome-wide significance (*P* = 5.3 × 10^−8^). Notably, all associations estimated in our replication study had the same direction of association as in the discovery analysis step.

**Table 3:** Replication results for the top NCadjBMI associations in Table 2.

				Sex-combined			Men			Women		
SNP	Chr	Gene	Alleles	*N*	Effect	*P*	*N*	Effect	*P*	*N*	Effect	*P*
rs227724	17	NOG	A/T	3296	−0.09	0.045	1554	−0.121	0.045	1740	−0.06	0.222
rs113515681	17	LOC105371884	T/G	3269	−0.07	0.344	1544	−0.168	0.235	1723	0.005	0.507
rs13087058	3	PDZRN3	T/C	3205	−0.088	0.049	1511	−0.084	0.128	1692	−0.094	0.108
rs10771543	12	OVCH1	A/G	3108	−0.056	0.145	1463	0.025	0.633	1643	−0.123	0.053
rs62101076	18	LOC101927404	A/G	3272	0.289	0.550	1537	0.489	0.025	1733	0.159	0.275
rs139177122	7	RN7SKP75	A/G	3257	−0.27	0.590	1537	−0.423	0.041	1720	−0.176	0.235

## Discussion

To the best of knowledge, this is the first genome-wide association analysis of NC, an index of upper-body fat that associates with multiple adverse metabolic traits and outcomes. In this multi-ethnic meta-analysis, we identified 16 genetic loci that show suggestive or significant association evidence with age and BMI-adjusted or unadjusted values of NC in sex-combined or sex-specific analyses. We tested six of the lead NCadjBMI SNPs in replication analysis in an independent study. Our analyses identified three genomic areas of particular interest: (a) a male-specific association for BMI-adjusted NC within an intergenic region between genes *NOG* and *C17orf67*, a locus which previously was associated with height; (b) a sex-combined association for BMI-adjusted NC with a SNP in gene *PDZRN3*, a locus previously associated with the ECG-derived PR interval; and (c) an association in BMI-unadjusted analyses of NC with a SNP in the *FTO* gene, which harbors multiple SNPs associated with energy intake and obesity [35]. Moreover, quantitative expression trait data show that these top SNPs are associated with gene expression in relevant tissues, including the esophagus (a major structure in the neck), subcutaneous adipose tissue, arteries, and musculo-skeletal tissue. These findings suggest that the genetic bases for NC overlap with genetic processes related to body size and neck anatomic structures, subcutaneous fat depots, and cardiac atrial conduction. Both *NOG* and *PDZRN3*—two genes identified by our top associations—interact with members of transforming growth factor-beta (TGF-β) superfamily signaling proteins, which are critical for processes related to somatic development and adipocyte biology, including regulation of the extracellular matrix [36] that can influence the patterning of fat depositions [37]. Therefore, the two genes may directly influence developmental processes associated with size of neck structures as well as fat deposition in the neck.

Our genome-wide significant SNP (rs227724) for BMI-adjusted NC emerged from an analysis in men. This SNP overlaps with an eQTL for the *NOG* gene, which encodes the Noggin protein. Noggin interferes with bone morphogenetic proteins (BMPs), members of the TGF-β superfamily that regulate cellular differentiation, proliferation, migration, and apoptosis [38], and influence a wide variety of tissue structures, most notably bone and cartilage, as well as the cardiovascular and adipose tissues [39]. In mice, the interaction between *NOG* and *BMP4* controls development of the axial skeleton and is critical for establishment and patterning of the somite morphogenesis [40]. *NOG* inhibits the differentiation of human stromal cells induced by *BMP4* [41], a protein induced by human precursor cells undergoing adipogenesis, playing an important role in regulating human white and brown adipose tissue [42]. Loss of Noggin inhibition of BMP4 signaling results in lipid accumulation and a “whitening” of brown adipose tissue [39], or a more severe metabolic phenotype. Of interest, BMP signaling, including interactions with *NOG*, in adipose tissue differs in male and female rodents, with female mice more responsive to Noggin depletion [39]. BMPs also are influential in the development of the embryonic foregut, which includes the neck structures, the esophagus, and trachea. Therefore, there are several strong plausible mechanisms underlying the association of this SNP with NC, including molecular processes influencing adipose tissues and neck anatomy.

The top SNP near *NOG* overlaps a height-associated locus from a previous GWAS [31], also suggesting that common underlying mechanisms determine height and NC. Prior studies have shown overlap between height-associated genetic variants and obesity variants [43]. Moreover, an analysis of over 400,000 individuals from the UK Biobank reported significant positive epidemiological and genetic associations between height with 11 diseases, including positive associations of height with atrial fibrillation and venous thromboembolism, and negative associations with coronary artery disease, and identified multiple molecular pathways linking these disorders, including TGF-β signaling [44]. Our analysis further identifies NC, likely reflecting subcutaneous upper-body fat, as an additional metabolic risk factor that is related to molecular mechanisms regulating body height.

The SNP rs13087058, associated with BMI-adjusted NC in sex-combined analysis, overlaps with an eQTL for the *PDZRN3* gene and is linked to decreased expression in esophageal and arterial tissues. *PDZRN3* encodes a member of the PDZ domain-containing RING finger family of proteins, which may function in the differentiation of adipocytes, osteoblasts, and myoblasts. *PDZRN3* also negatively regulates the differentiation of C2C12 mouse mesenchymal progenitor cells into BMP-2-induced osteoblasts [45] and adipogenesis in 3T3-L1 mouse cells [46], as well as influences endothelial cell junction stability and vascular integrity [47]. The SNP rs13087058 also was previously identified to associate with ECG-derived PR interval [32]. PR interval reflects the properties of electrical conduction between the atria and ventricles, with short or long PR interval associated with an increased incidence of atrial fibrillation [48] as well as cardiovascular mortality [49]. Although the relationship between NC and cardiac atrial disease is not clear, these conditions may be linked via cardiometabolic risk factors. Alternatively, both may be related to aspects of body growth. Each ~6-cm increase in standing height is associated with a 30–40% increased odds of atrial fibrillation [44]. It has been postulated that factors promoting body growth also promote atrial growth, which influences its electrical phenotype. It is intriguing that both of the top NC SNPs are associated with body height via direct or indirect (i.e., via atrial fibrillation) relationships, and both associations implicate genes in the TGF-β family.

The genome-wide significant SNP (rs56094641) for NC without adjusting for BMI is an intron variant in the *FTO* gene. *FTO* is a widely replicated obesity gene and is associated with a variety of obesity traits across diverse ancestries [35]. Multiple SNPs in introns 1 and 2 of *FTO* remain to be among the loci with largest effect sizes on BMI. A pathway for adipocyte thermogenesis regulation has been found to elucidate the potential mechanic basis for the genetic association between *FTO* and obesity [50]. Our finding suggests that the *FTO* gene may also affect the fat in the neck, perhaps by increasing overall body fat.

Increasing NC is associated with a higher prevalence of OSA, a common disorder associated with central obesity, diabetes, hypertension, cardiovascular disease, and atrial fibrillation [51]. The relationship between NC and sleep apnea is potentially complex, with a larger neck size reflecting more fat around and within the upper airway, resulting in narrowing of the airway caliber and increased airway collapsibility, with subsequent airway collapse (manifest as hypopneas and apneas) [52, 53]. In addition, sleep apnea risk may be increased in individuals with a larger NC due to more generalized effects of central obesity in reducing lung volumes. A large NC also could reflect an altered metabolic phenotype that increases sleep apnea risk via generalized inflammation and insulin resistance [54], which can influence local fat metabolism as well as effect respiratory control. In a look up in published summary statistics, we observed strong association between our top *NOG* SNP and snoring (a cardinal symptom of OSA), in analyses performed in a large study (*N* = ~407K) of individuals of European ancestry. However, we observed only weak to modest associations between NC-associated SNPs and several, more specific, OSA traits (such as measures of oxygen saturation during sleep and the apne–hypopnea index) in the smaller available GWAS of quantitative OSA traits. However, due to the modest sample sizes for these analyses, they are likely under-powered to detect small to modest associations. Future, large studies, of NC and measures of OSA are needed to study the effect of the *NOG* gene on the upper airway and the potential mediating role of NC as a potential causal mechanism for OSA.

Our findings support prior GWAS findings that there are sex-specific genetic loci for body-fat distribution. The GIANT consortium identified 44 loci with significant sex differences for waist-to-hip ratio adjusted for BMI, of which 28 showed more prominent effects in women than in men, 5 showed prominent effects in men than in women, and 11 showed opposite effect between men and women [14]. Similarly, a locus at *THNSL2* was uncovered in association with visceral adipose tissue only in women, but not in men [55]. Recent GWAS based on the individuals from the UK biobank also identified sex-specific loci that are associated with the proportion of fat in legs and trunk [56]. The results from our analysis extend these observations to NC, a measure of body-fat distribution that is associated cardiovascular risk factors independently of BMI and waist circumference [17, 20, 21]. In our results, several genetic associations are stronger in men than those in women for NC both adjusted and unadjusted for BMI. Our most significant association was observed for BMI-adjusted NC in men with a SNP near *NOG*. As described earlier, this pathway for adipocyte thermogenesis has sex-specific differences in mice [39]. However, when interpreting sex differences, it is also important to note that data from the FHS showed stronger correlations between measured NC and imaging-based upper-body subcutaneous fat measurements in men (*r* = 0.61–0.77) compared to women (*r* = 0.33–0.39) [17], consistent with more measurement error in women, which may attenuate true associations.

While we studied sex differences in the genetic basis of NC and NCadjBMI, we were not able to study ancestry-based differences, due to low sample size of AAs (*N* = 1465 males and female combined). Previous GWAS of BMI and of a related phenotype, metabolic syndrome, identified African ancestry-specific variant associations [57, 58], in agreement with reports showing higher subcutaneous adipose and lower visceral adipose tissue in AAs compared to EAs [59, 60]. Further research is needed to better understand potential ancestry-specific associations in additional studies of body-fat distribution, including of NC and NCadjBMI.

In summary, we identified a significant male-specific novel locus near the *NOG* gene for NCadjBMI and an intronic variant in the *FTO* gene for NC at the genome-wide significance level, and several loci at the suggestive significance level for NCadjBMI or NC, including a SNP near *PDZRN3*. eQTL analyses identified altered gene expression with the lead SNPs in relevant tissues, including the esophagus, subcutaneous tissue, and arteries. Associations also implicated *NOG* and *PDZRN3*, both of which interact with the TGF-β family of growth factors. Sex-stratified analysis also suggested that the genetic effects for NC clearly differ between men and women, consistent with the previous findings for other measurements of body-fat distribution. As NC was reported to be uniquely associated with OSA and cardiometabolic diseases, understanding the genetic mechanisms and determinants for the fat deposited around neck may help in assessing the risk of these diseases. However, further GWAS with a large sample sizes of men and women are warranted for replications and fine-mapping of loci associated with NC.

## Supplementary information

Supplementary Materials

## Data Availability

Summary statistics from GWAS of NC and NCadjBMI will be made available on the GWAS Catalog https://www.ebi.ac.uk/gwas/ and on the Sleep Disorders Knowledge Portal https://sleep.hugeamp.org/.
