# Supplementary material for: Genome-wide association study of neck circumference identifies sex-specific loci independent of generalized adiposity
Source: Int J Obes (Lond). 2021 Apr 27;45(7):1532–41. doi: 10.1038/s41366-021-00817-2 (PMC8236408; doi:10.1038/s41366-021-00817-2)

Genome-Wide Association Study of Neck Circumference Identifies Sex-Specific Loci Independent of Generalized Adiposity: Supplementary Information

Yaowu Liu, Xiaoyu Zhang, Jiwon Lee, Diane Smelser, Brian Cade, Han Chen,
Hufeng Zhou, H. Lester Kirchner, Xihong Lin, Sutapa Mukherjee, David Hillman,
Ching-Ti Liu, Susan Redline, Tamar Sofer

[Supplementary Methods 1](#_Toc65933678)

[Primary Samples: Discovery Cohorts 1](#_Toc65933679)

[The Framingham Heart Study (FHS) 1](#_Toc65933680)

[The Sleep Heart Health Study 2](#_Toc65933681)

[The Cardiovascular Health Study (CHS): 3](#_Toc65933682)

[The Atherosclerosis Risk In Communities (ARIC) study 4](#_Toc65933683)

[The Cleveland Family Study (CFS): 4](#_Toc65933684)

[The Osteoporotic Fractures in Men Study (MrOS) Sleep Study: 5](#_Toc65933685)

[The Jackson Heart Study (JHS): 6](#_Toc65933686)

[The Western Australian Sleep Health Study (WASHS): 7](#_Toc65933687)

[The Geisinger dataset: Generalizability/Replication 7](#_Toc65933688)

[Geisinger Sleep Lab information: 8](#_Toc65933689)

[Statistical Analysis 9](#_Toc65933690)

[Primary Analysis 9](#_Toc65933691)

[Replication Analysis 9](#_Toc65933692)

[References 10](#_Toc65933693)

[Supplemental Tables 12](#_Toc65933694)

[Supplemental Figures 24](#_Toc65933695)

# Supplementary Methods

## Primary Samples: Discovery Cohorts

### The Framingham Heart Study (FHS)

FHS is a community-based cohort study that started in 1948, aiming to identify the factors that contribute to coronary vascular disease and other major illness. The Original Cohort included 5,209 men and women between the ages of 28 and 62 years from Framingham, MA, USA(1). The children and the children’s spouses (Offspring Cohort: 5124 participants) and the grandchildren (Third Generation Cohort: 4095 participants) of the original cohort were also enrolled in the FHS later. FHS participants underwent physical examination, medical history, and routine laboratory tests approximately every two or four years. Individuals with genotype information, neck circumference measurements and relevant covariates from the Offspring Cohort (at exam 6) and the Third Generation Cohort (at exam 1), in total of 6950 participants, were included for this study. Neck circumference was measured by an anthropometric tape. The superior border of the tape was placed inferior to the laryngeal prominence and the tap was applied snugly perpendicular to the long axis of the neck. Neck circumference was recorded to the nearest ¼ inch, rounding down, and was multiplied by 2.54 as centimeters. All participants provided written informed consent and the study protocol was approved by the Boston University Medical Campus institutional review boards.

### The Sleep Heart Health Study

The Sleep Heart Health Study (SHHS) was a community-based study designed to investigate the association between sleep disordered breathing and cardiovascular disease as described before(2). In brief, participants ages 40 years and older were recruited from ongoing cohort studies (“parent” cohorts) and were asked to participate in a baseline sleep examination between 1995 and 1998 which consisted of in-home polysomnography (PSG), anthropometry, including measurement of Neck Circumference, blood pressure measurement, and questionnaire completion. Of the “parent” cohorts that participated in SHHS and therefore had measurements of Neck Circumference, 3 also had genotyping data and are included in the current analysis: ARIC, CHS, and FHS. However, for FHS, NC measurements not taken from SHHS. The institutional review boards of all participating institutions approved the study, and participants signed a consent form.

### The Cardiovascular Health Study (CHS):

CHS is a population-based cohort study of risk factors for coronary heart disease and stroke in adults ≥65 years conducted across four field centers(3). The original predominantly European ancestry cohort of 5,201 persons was recruited in 1989- 1990 from random samples of the Medicare eligibility lists; subsequently, an additional predominantly African-American cohort of 687 persons were enrolled for a total sample of 5,888. Blood samples were drawn from all participants at their baseline examination and DNA was subsequently extracted from available samples. Genotyping was performed at the General E8 Clinical Research Center’s Phenotyping/Genotyping Laboratory at Cedars-Sinai among CHS participants who consented to genetic testing and had DNA available using the Illumina 370CNV BeadChip system (for European ancestry participants, in 2007) or the Illumina HumanOmni1- Quad_v1 BeadChip system (for African-American participants, in 2010). Additional genotypes were provided from the ITMAT-Broad-CARe (IBC) Illumina iSELECT chip. A subset of CHS participants also participated in the SHHS described above, and were measured neck circumference.

**CHS acknowledgements:** This research was supported by contracts HHSN268201200036C, HHSN268200800007C, HHSN268201800001C, N01HC55222, N01HC85079,

N01HC85080, N01HC85081, N01HC85082, N01HC85083, N01HC85086, and grants

U01HL080295 and U01HL130114 from the National Heart, Lung, and Blood Institute

(NHLBI), with additional contribution from the National Institute of Neurological

Disorders and Stroke (NINDS). Additional support was provided by R01AG023629 from

the National Institute on Aging (NIA). A full list of principal CHS investigators and

institutions can be found at [CHS-NHLBI.org](https://urldefense.proofpoint.com/v2/url?u=http-3A__CHS-2DNHLBI.org&d=DwMGaQ&c=WO-RGvefibhHBZq3fL85hQ&r=axNGbXAq7l67SLduYW4ARp09GJ8e8B4u3_IV68vZHC0&m=WleDE9aTKPgavpgHCvEf3l4hYxkOU-xPWwRlLnXzVKg&s=2o2fb0D_PVsWjft8eKM4YZkHsV57ZMRGYla5wubR7Ww&e=). Support for the genotyping through the CARe Study was provided by NHLBI Contract N01HC65226.

### The Atherosclerosis Risk In Communities (ARIC) study

ARIC is a population-based prospective cohort study of cardiovascular disease, following 15,792 individuals aged 45-64 years at baseline (1987-89), selected into the study by probability sampling from four US communities(4). Neck Circumference measures and other phenotypes were taken from the SHHS, described above.

**ARIC acknowledgements:** The Atherosclerosis Risk in Communities Study is carried out as a collaborative study supported by National Heart, Lung, and Blood Institute contracts N01-HC-55015, N01-HC-55016, N01-HC-55018, N01-HC-55019, N01-HC-55020, N01-HC-55021, N01-HC-55022, R01HL087641, R01HL59367 and R01HL086694; National Human Genome Research Institute contract U01HG004402; and National Institutes of Health contract HHSN268200625226C. The authors thank the staff and participants of the ARIC study for their important contributions. Infrastructure was partly supported by Grant Number UL1RR025005, a component of the National Institutes of Health and NIH Roadmap for Medical Research.

### The Cleveland Family Study (CFS):

CFS is a family-based longitudinal study designed to examine the genetic basis of sleep apnea in African-Americans and European-Americans. Index probands with confirmed sleep apnea were recruited in 1990 from sleep centers in Northern Ohio, along with additional family members and neighborhood control families(5). In all, 356 families participated in the study. Over four waves of data collection over 16 years (last exam in 2016), individuals underwent measurements for sleep apnea, anthropometry, and other related phenotypes. Neck circumference was measured in all exams, we used the last available measure. Institutional Review Board approval and signed informed consent was obtained for all participants.

**CFS acknowledgements:** Support for the Cleveland Family Study was provided by NHLBI grant number R01 HL46380 and NHLBI grant number R01 HL113338.

### The Osteoporotic Fractures in Men Study (MrOS) Sleep Study:

MrOS(6, 7) is a prospective cohort of 5,994 men that were age 65 or older recruited between 2000 and 2002 in six communities: Birmingham AL, Minneapolis MN, Palo Alto CA, Monongahela Valley PA, Portland OR, and San Diego CA. Neck circumference was measured in the ancillary MrOS Sleep Study. This ancillary study was conducted between December 2003 and March 2005 and recruited 3135 of these participants who were not regular users of CPAP and did not use overnight oxygen or oral appliances to participate in overnight in-home polysomnography, anthropometry, questionnaire completion and other measures, as described before(8). The protocol was approved by the institutional review boards at all of the participating institutions and all participants provided written informed consent. Of these individuals, 2,364 self-reported European Americans were genotyped. Subsequently, 138 participants with missing BMI and/or Neck Circumference and 17 ancestry outliers from principal component analysis(9) were excluded, resulting in 2195 participants included in the analysis.

**MrOS acknowledgements:** The Osteoporotic Fractures in Men (MrOS) Study is supported by National Institutes of Health funding. The following institutes provide support: the National Institute on Aging (NIA), the National Institute of Arthritis and Musculoskeletal and Skin Diseases (NIAMS), the National Center for Advancing Translational Sciences (NCATS), and NIH Roadmap for Medical Research under the following grant numbers: U01 AG027810, U01 AG042124, U01 AG042139, U01 AG042140, U01 AG042143, U01 AG042145, U01 AG042168, U01 AR066160, and UL1 TR000128. The National Heart, Lung, and Blood Institute (NHLBI) provides funding for the MrOS Sleep ancillary study "Outcomes of Sleep Disorders in Older Men" under the following grant numbers: R01 HL071194, R01 HL070848, R01 HL070847, R01 HL070842, R01 HL070841, R01 HL070837, R01 HL070838, and R01 HL070839. The National Institute of Arthritis and Musculoskeletal and Skin Diseases (NIAMS) provides funding for the MrOS ancillary study ‘GWAS in MrOS and SOF’ under the grant number RC2 AR058973.

### The Jackson Heart Study (JHS):

JHS is a large, population-based observational study evaluating the etiology of cardiovascular diseases and related disorders among African Americans residing in the three counties (Hinds, Madison, and Rankin) that constitute the Jackson, Mississippi metropolitan area(10). Data and biologic materials have been collected from 5,301 participants, including a nested family cohort of 1,498 members of 264 families. The age at enrollment for the unrelated cohort was 35-84 years; the family cohort included related individuals >21 years old. During a baseline examination (2000-2004) and two follow-up examinations (2005-2008 and 2009-2012), participants provided extensive medical and social history, had an array of physical and biochemical measurements and diagnostic procedures, and provided blood for genomic DNA(11). The study population is characterized by a high prevalence of diabetes, hypertension, obesity, and related disorders. Annual follow-up interviews and cohort surveillance are ongoing.

The current paper uses data from the JHS Sleep ancillary study (JHSS), which was conducted between December 2012 and May 2016 after the third examination. Institutional review board approval was obtained from the University of Mississippi and Partners Research Committee, and written informed consent was obtained from all participants. Neck circumference was measured as part of the JHSS by trained staff.

**JHS acknowledgments:** The Jackson Heart Study (JHS) is supported and conducted in collaboration with Jackson State University (HHSN268201800013I), Tougaloo College (HHSN268201800014I), the Mississippi State Department of Health (HHSN268201800015I/HHSN26800001) and the University of Mississippi Medical Center (HHSN268201800010I, HHSN268201800011I and HHSN268201800012I) contracts from the National Heart, Lung, and Blood Institute (NHLBI) and the National Institute for Minority Health and Health Disparities (NIMHD). The authors also wish to thank the staffs and participants of the JHS. The JHSS was supported by grant R01HL110068 from NHLBI.

**JHS Disclaimer**: The views expressed in this manuscript are those of the authors and do not necessarily represent the views of the National Heart, Lung, and Blood Institute; the National Institutes of Health; or the U.S. Department of Health and Human Services

### The Western Australian Sleep Health Study (WASHS):

WASHS is a clinic-based study designed to discover the genetics of obstructive sleep apnea and its comorbidities(12). The study examines individuals presenting to the public sleep clinic in Perth, Western Australia. Data collection for patients tested in the current study occurred from 2006 – 2010. Most (91% of patients) were referred to the sleep clinic for OSA. Sleep, anthropometric, and other phenotypes were measured, as well as biochemical measures. In this study we considered 1,960 individuals who consented for genotyping and had Neck Circumference measurements. We excluded principal component outliers, resulting in 1,582 individuals of European ancestry.

## The Geisinger dataset: Generalizability/Replication

Geisinger is a large integrated health system serving patients across 45 of 67 counties in Pennsylvania, as well as parts of New Jersey. As an integrated system, Geisinger incorporates within a single not-for-profit institution a large primary care and specialty group practice, more than 70 care sites (including 2 tertiary-quaternary care hospitals and other inpatient facilities and a network of community-based clinics), and an insurance operation. This integration creates a more seamless approach to care and more complete capture of episodes of care. Much of the population served by Geisinger is relatively non-transient, with low rates of migration into or out of the area, a large number of life-long residents, and many multi-generation families. Geisinger was an early adopter of electronic health record (EHR) systems (beginning in 1996); its EHR is fully implemented across all sites of care. In 2007 Geisinger launched the MyCode® Community Health Initiative (13)(MyCode®) to create a system-wide biorepository of blood, serum and DNA samples for broad research use, including genomic analysis. Data obtained from analysis of MyCode® samples can be linked to information in participants’ digital health records.

### Geisinger Sleep Lab information:

The Geisinger Sleep Lab is a clinically referred population with high referrals from Cardiology, Pulmonary and Neurology. Patients are referred for overnight laboratory polysomnography recordings included an electroencephalogram (C4A1, Fp1A2, O1A2), two channels of electro-oculogram (EOG), submental electromyogram (EMG), bilateral anterior tibial electromyogram, electrocardiogram, summed oronasal airflow by thermocouple, thoracic and abdominal movement by inductive plethysmography, and pulse oximetry. In-lab PSG were collected on one of three computerized recording systems (Harmonie-S, Stellate, Montreal, Canada; Sandman, Natus Medical Inc, Middleton, Wisconsin; Alice 6 LDxS, Philips Respironics, Murrysville, Pennsylvania). All interpretation and scoring performed manually by experienced sleep technicians, then reviewed and revised by sleep medicine physicians. Sleep parameters was scored on screen by conventional criteria(8, 14-16) (Rechtschaffen A, Kales A, 1968; Iber et al., 2007; Berry et al, 2012; Berry et al., 2017) using the ≥4% oxygen desaturation criteria for hypopnea. Studies without four hours of interpretable recording were considered technically unsatisfactory and not included in this study. Neck circumference is measured at the consultation visit with the sleep medicine physician. There is not specific protocol followed to do the measurement other than it is taken at the shirt collar the patient is wearing and recorded in inches. Then converted to cm for analysis.

## Statistical Analysis

### Primary Analysis

Neck circumference residuals after adjusting for BMI were constructed and were used as the phenotype NCadjBMI. Within each discovery cohort and ancestral background, NC and NCadjBMI were adjusted for age and sex (age only in the all-male MrOS cohort), and the first 3 PCs. Linear mixed models including a genetic relatedness matrix (GRM) calculated as the cross-product of the standardized genome-wide genotype matrix were applied using GEMMA(17) to control for population stratification and relatedness in all the cohort. Multi-ethnic meta-analyses were performed using the inverse variance weighted fixed-effects approach, using METAL(18). Sex-specific analyses were conducted using the linear mixed model as described above. SNPs present with a combined sample size less than 2,000 were excluded after meta analyses. Statistical significance was defined as P < 5.0 × 10^-8^. We also report SNPs with suggestive significance of P<1x10^-6^. Discovery analysis p-values are two-sided.

### Replication Analysis

Patients with sleep study data used for the replication analysis were extracted from the DiscovEHR dataset. The genotyping for this dataset was performed in two stages. The first 57,118 samples were chipped on the Illumina HumanOmniExpressExome/InfiniumOmniExpressExome BeadChip, three versions were used across the samples, v1.2, 1.3 and 1.4. The remaining 31,063 samples were typed on the Illumina Infinium Global Screening Array-24 v1.0 (GSA) BeadChip. Calling was performed with Genome Analysis Toolkit software. Quality control thresholds included a genotyping call rate of 99% and MAF>1%. SNPs with a significant batch effect were removed (n=113). Individuals with a sample call rate of < 90% were removed. Principal components were generated using PLINK 2.0. A total of 632,574 variants were used for imputation, with SHAPEIT and IMPUTE2 used for pre-phasing and imputation. 1000 Genomes (GRCh37) was used as the reference panel. Post-Imputation QC included Info score ≥0.3, MAF ≥0.01, and HWE >1e-6. Plink was used to perform linkage disequilibrium pruning, using 1000 as the window size, 50 for the step size and r2 threshold of 0.2. The variants were tested for association with the neck circumference phenotype using linear regression in Plink. Covariates included in the model were sex, age, BMI, and the first 4 PCs.

## References

1. Dawber TR, Meadors GF, Moore Jr FE. Epidemiological approaches to heart disease: the Framingham Study. American Journal of Public Health and the Nations Health. 1951;41(3):279-86.

2. Quan SF, Howard BV, Iber C, Kiley JP, Nieto FJ, O'Connor GT, et al. The sleep heart health study: design, rationale, and methods. Sleep. 1997;20(12):1077-85.

3. Fried LP, Borhani NO, Enright P, Furberg CD, Gardin JM, Kronmal RA, et al. The cardiovascular health study: design and rationale. Annals of epidemiology. 1991;1(3):263-76.

4. Investigators A. The atherosclerosis risk in communit (aric) stui) y: Design and objectwes. American journal of epidemiology. 1989;129(4):687-702.

5. Redline S, Tishler PV, Tosteson TD, Williamson J, Kump K, Browner I, et al. The familial aggregation of obstructive sleep apnea. American journal of respiratory and critical care medicine. 1995;151(3_pt_1):682-7.

6. Orwoll E, Blank JB, Barrett-Connor E, Cauley J, Cummings S, Ensrud K, et al. Design and baseline characteristics of the osteoporotic fractures in men (MrOS) study—a large observational study of the determinants of fracture in older men. Contemporary clinical trials. 2005;26(5):569-85.

7. Blank JB, Cawthon PM, Carrion-Petersen ML, Harper L, Johnson JP, Mitson E, et al. Overview of recruitment for the osteoporotic fractures in men study (MrOS). Contemporary clinical trials. 2005;26(5):557-68.

8. Mehra R, Stone KL, Blackwell T, Ancoli Israel S, Dam TTL, Stefanick ML, et al. Prevalence and correlates of sleep‐disordered breathing in older men: osteoporotic fractures in men sleep study. Journal of the American Geriatrics Society. 2007;55(9):1356-64.

9. Wang C, Zhan X, Liang L, Abecasis GR, Lin X. Improved ancestry estimation for both genotyping and sequencing data using projection procrustes analysis and genotype imputation. The American Journal of Human Genetics. 2015;96(6):926-37.

10. Taylor Jr HA, Wilson JG, Jones DW, Sarpong DF, Srinivasan A, Garrison RJ, et al. Toward resolution of cardiovascular health disparities in African Americans: design and methods of the Jackson Heart Study. Ethn Dis. 2005;15(4 Suppl 6):S6-4.

11. Wilson JG, Rotimi CN, Ekunwe L, Royal C, Crump ME, Wyatt SB, et al. Study design for genetic analysis in the Jackson Heart Study. Ethnicity & disease. 2005;15(4 Suppl 6):S6-30.

12. Mukherjee S, Hillman D, Lee J, Fedson A, Simpson L, Ward K, et al. Cohort profile: the Western Australian sleep health study. Sleep and Breathing. 2012;16(1):205-15.

13. Carey DJ, Fetterolf SN, Davis FD, Faucett WA, Kirchner HL, Mirshahi U, et al. The Geisinger MyCode community health initiative: an electronic health record–linked biobank for precision medicine research. Genetics in medicine. 2016;18(9):906-13.

14. Hobson JA. A manual of standardized terminology, techniques and scoring system for sleep stages of human subjects: A. Rechtschaffen and A. Kales (Editors).(Public Health Service, US Government Printing Office, Washington, DC, 1968, 58 p., $4.00). Elsevier; 1969.

15. Berry RB, Brooks R, Gamaldo CE, Harding SM, Marcus C, Vaughn BV. The AASM manual for the scoring of sleep and associated events. Rules, Terminology and Technical Specifications, Darien, Illinois, American Academy of Sleep Medicine. 2012;176:2012.

16. Berry RB, Brooks R, Gamaldo C, Harding SM, Lloyd RM, Quan SF, et al. AASM scoring manual updates for 2017 (version 2.4). American Academy of Sleep Medicine; 2017.

17. Zhou X, Stephens M. Genome-wide efficient mixed-model analysis for association studies. Nature genetics. 2012;44(7):821-4.

18. Willer CJ, Li Y, Abecasis GR. METAL: fast and efficient meta-analysis of genomewide association scans. Bioinformatics. 2010;26(17):2190-1.

19. Campos AI, García-Marín LM, Byrne EM, Martin NG, Cuéllar-Partida G, Rentería ME. Insights into the aetiology of snoring from observational and genetic investigations in the UK Biobank. Nature Communications. 2020;11(1):817.

# Supplemental Tables

**Supplement Table 1:** Complete significance and suggestive meta-analysis result for NCadjBMI and NC. Lead SNPs with p-value < 1x10^-6^ in at least one of the analyses are reported.

|  |  |  |  |  | **NCadjBMI** | | **NC** | |
| --- | --- | --- | --- | --- | --- | --- | --- | --- |
|  | **SNP** | **Chr** | **Alleles** | **N** | **Effect** | **P** | **Effect** | **P** |
| **Sex-combined** | rs227724 | 17 | A/T | 15090 | -0.1367 | 6.59E-08 | -0.1403 | 5.38E-05 |
|  | rs113515681 | 17 | T/G | 13625 | -0.4338 | 1.59E-07 | -0.4327 | 1.45E-04 |
|  | rs13087058 | 3 | T/C | 15090 | -0.1288 | 2.94E-07 | -0.1463 | 2.29E-05 |
|  | rs10771543 | 12 | A/G | 15090 | -0.1193 | 8.29E-07 | -0.0639 | 5.61E-02 |
|  | rs62101076 | 18 | A/G | 13625 | 0.4325 | 9.52E-07 | 0.3346 | 6.54E-03 |
|  | rs139177122 | 7 | A/G | 13625 | -0.4093 | 2.60E-06 | -0.4821 | 4.12E-05 |
|  | rs56094641 | 16 | A/G | 15090 | -0.0339 | 1.69E-01 | -0.2043 | 1.48E-09 |
|  | rs12422456 | 12 | A/C | 13625 | -0.1123 | 7.82E-03 | -0.3082 | 1.27E-07 |
|  | rs4606526 | 12 | A/G | 13831 | -0.155 | 2.03E-04 | -0.287 | 5.41E-07 |
|  | rs34342452 | 19 | C/G | 13831 | -0.08 | 7.95E-03 | -0.1679 | 4.76E-05 |
|  | rs12647166 | 4 | A/T | 14366 | 0.0326 | 1.82E-01 | 0.0526 | 1.19E-01 |
|  | rs10244704 | 7 | T/C | 15090 | -0.0599 | 7.13E-02 | -0.1176 | 1.03E-02 |
|  | rs1483197 | 1 | T/C | 14366 | -0.0792 | 4.02E-03 | -0.1376 | 1.98E-04 |
|  | rs7647178 | 3 | T/G | 15090 | -0.1077 | 1.66E-05 | -0.154 | 7.96E-06 |
|  | rs12823146 | 12 | T/G | 13831 | 0.0873 | 8.65E-04 | 0.141 | 8.75E-05 |
| **Men** | rs227724 | 17 | A/T | 8331 | -0.1985 | 1.74E-09 | -0.1825 | 1.94E-05 |
|  | rs113515681 | 17 | T/G | 7430 | -0.4828 | 7.93E-06 | -0.5725 | 4.82E-05 |
|  | rs13087058 | 3 | T/C | 8331 | -0.139 | 1.76E-05 | -0.1824 | 1.41E-05 |
|  | rs10771543 | 12 | A/G | 8331 | -0.1152 | 2.49E-04 | -0.0607 | 1.41E-01 |
|  | rs62101076 | 18 | A/G | 7430 | 0.3377 | 3.62E-03 | 0.2882 | 6.26E-02 |
|  | rs139177122 | 7 | A/G | 7430 | -0.5769 | 3.67E-07 | -0.8301 | 9.39E-09 |
|  | rs56094641 | 16 | A/G | 8331 | -0.0413 | 1.94E-01 | -0.1956 | 1.99E-06 |
|  | rs12422456 | 12 | A/C | 7730 | -0.0787 | 1.51E-01 | -0.2778 | 1.05E-04 |
|  | rs4606526 | 12 | A/G | 7730 | -0.1546 | 4.17E-03 | -0.2804 | 6.34E-05 |
|  | rs34342452 | 19 | C/G | 7812 | -0.0533 | 1.68E-01 | -0.0573 | 2.54E-01 |
|  | rs12647166 | 4 | A/T | 8014 | -0.0414 | 1.92E-01 | -0.0725 | 8.28E-02 |
|  | rs10244704 | 7 | T/C | 8331 | -0.0037 | 9.32E-01 | 0.0321 | 5.75E-01 |
|  | rs1483197 | 1 | T/C | 8014 | -0.1406 | 6.21E-05 | -0.2364 | 1.29E-07 |
|  | rs7647178 | 3 | T/G | 8331 | -0.1437 | 1.02E-05 | -0.2208 | 1.97E-07 |
|  | rs12823146 | 12 | T/G | 7812 | 0.1391 | 4.19E-05 | 0.224 | 3.02E-07 |
| **Women** | rs227724 | 17 | A/T | 6768 | -0.0643 | 7.82E-02 | -0.082 | 1.49E-01 |
|  | rs113515681 | 17 | T/G | 5896 | -0.3187 | 8.02E-03 | -0.1537 | 4.31E-01 |
|  | rs13087058 | 3 | T/C | 6768 | -0.1089 | 3.05E-03 | -0.0737 | 2.02E-01 |
|  | rs10771543 | 12 | A/G | 6768 | -0.1298 | 2.10E-04 | -0.0583 | 2.84E-01 |
|  | rs62101076 | 18 | A/G | 5437 | 0.5491 | 3.16E-05 | 0.3066 | 1.64E-01 |
|  | rs139177122 | 7 | A/G | 5531 | -0.1511 | 2.54E-01 | 0.2099 | 3.14E-01 |
|  | rs56094641 | 16 | A/G | 6768 | -0.0213 | 5.53E-01 | -0.2389 | 2.17E-05 |
|  | rs12422456 | 12 | A/C | 5896 | -0.1608 | 8.52E-03 | -0.4091 | 3.11E-05 |
|  | rs4606526 | 12 | A/G | 6020 | -0.1438 | 1.84E-02 | -0.3131 | 1.37E-03 |
|  | rs34342452 | 19 | C/G | 6020 | -0.1254 | 4.71E-03 | -0.3684 | 2.23E-07 |
|  | rs12647166 | 4 | A/T | 6361 | 0.1517 | 1.71E-05 | 0.2844 | 3.07E-07 |
|  | rs10244704 | 7 | T/C | 6768 | -0.1661 | 4.55E-04 | -0.3606 | 7.40E-07 |
|  | rs1483197 | 1 | T/C | 6361 | 0.0511 | 2.13E-01 | 0.0528 | 4.13E-01 |
|  | rs7647178 | 3 | T/G | 6768 | -0.0499 | 1.67E-01 | -0.0304 | 5.90E-01 |
|  | rs12823146 | 12 | T/G | 6020 | -0.0042 | 9.11E-01 | -0.0356 | 5.52E-01 |

**Supplement Table 2:** P-values of our top NC or NCadjBMI SNPs for other traits from the cardiovascular disease portal(<http://www.broadcvdi.org/>), the type 2 diabetes portal (<http://www.type2diabetesgenetics.org/>), and the GWAS catalog (<https://www.ebi.ac.uk/gwas/>).

| **Trait** | **P-values** | | |
| --- | --- | --- | --- |
|  | **rs227724** | **rs13087058** | **rs56094641** |
| Atrial fibrillation | 8.43E-02 | 4.83E-01 | 1.32E-03 |
| Bipolar disorder | 8.09E-01 | 4.33E-01 | NA |
| BMI | 4.12E-01 | 8.71E-01 | NA |
| Cholesterol | 4.49E-01 | 6.99E-01 | 2.04E-01 |
| Chronic kidney disease | 1.50E-01 | 8.10E-01 | NA |
| Disposition index | 8.60E-01 | 5.16E-01 | NA |
| Fasting glucose | 7.30E-01 | 5.66E-01 | NA |
| Fasting insulin | 7.21E-01 | 8.97E-01 | NA |
| HbA1c | 8.40E-01 | 4.97E-01 | NA |
| HDL cholesterol | 9.61E-01 | 1.50E-02 | 1.99E-03 |
| Heart failure | 9.58E-01 | 2.76E-01 | 5.79E-01 |
| Height | **3.50E-21** | 4.80E-01 | NA |
| HOMA-B | 9.36E-02 | 6.29E-01 | NA |
| HOMA-IR | 3.21E-01 | 9.82E-01 | NA |
| LDL cholesterol | 5.14E-01 | 7.19E-01 | 8.27E-01 |
| Major depressive disorder | 4.60E-01 | 5.92E-01 | NA |
| PR interval | 6.53E-01 | **5.82E-17** | NA |
| Schizophrenia | 7.00E-01 | 1.13E-01 | NA |
| Triglycerides | 3.48E-01 | 9.11E-01 | 1.29E-04 |
| Type 2 diabetes | 1.70E-01 | 4.10E-01 | **5.90E-25** |
| Type 2 diabetes adj BMI | 9.90E-01 | 6.90E-01 | NA |
| Waist circumference | 2.42E-04 | 9.67E-01 | NA |
| Waist-hip ratio | 8.78E-03 | 5.30E-01 | NA |

**Supplement Table 3:** P-values of our top NC or NCadjBMI SNPs for traits related to obstructive sleep apnea from the sleep disorders knowledge portal (http://sleepdisordergenetics.org/).

| **BMI-adjusted Phenotypes** | **SNP** | **Chromosome** | **Beta** | **StdErr** | **P** | **N** |
| --- | --- | --- | --- | --- | --- | --- |
| avg_desat | rs10771543 | 12 | -0.0005 | 0.0217 | 0.9806 | 10060 |
| avg_desat_females | rs10771543 | 12 | -0.0058 | 0.0267 | 0.8274 | 4117 |
| avg_desat_males | rs10771543 | 12 | 0.0075 | 0.0303 | 0.8046 | 5943 |
| avg_sao2 | rs10771543 | 12 | -0.0174 | 0.0122 | 0.155 | 21127 |
| avg_sao2_females | rs10771543 | 12 | -0.0247 | 0.0145 | 0.08945 | 10647 |
| avg_sao2.males | rs10771543 | 12 | -0.0143 | 0.0194 | 0.4605 | 10480 |
| min_sao2 | rs10771543 | 12 | -0.0188 | 0.0612 | 0.7583 | 21128 |
| min_sao2.females | rs10771543 | 12 | -0.0987 | 0.0827 | 0.2328 | 10652 |
| min_sao2.males | rs10771543 | 12 | 0.0373 | 0.088 | 0.6714 | 10476 |
| percent_sleep_under_90_saturation | rs10771543 | 12 | 0.0979 | 0.0442 | 0.02673 | 21260 |
| percent_sleep_under_90_saturation.females | rs10771543 | 12 | 0.0911 | 0.0435 | 0.03635 | 10714 |
| percent_sleep_under_90_saturation_males | rs10771543 | 12 | 0.0809 | 0.0799 | 0.3109 | 10546 |
| rdi_3p | rs10771543 | 12 | 0.1296 | 0.1332 | 0.3304 | 21244 |
| rdi_3p_females | rs10771543 | 12 | 0.2392 | 0.15 | 0.1107 | 10697 |
| rdi_3p_males | rs10771543 | 12 | 0.0646 | 0.2148 | 0.7636 | 10547 |
| avg_desat | rs113515681 | 17 | 0.0701 | 0.0725 | 0.3342 | 8288 |
| avg_desat_females | rs113515681 | 17 | 0.0115 | 0.0969 | 0.9059 | 3089 |
| avg_desat_males | rs113515681 | 17 | 0.0929 | 0.0967 | 0.337 | 5199 |
| avg_sao2 | rs113515681 | 17 | -0.0146 | 0.0384 | 0.7038 | 18984 |
| avg_sao2_females | rs113515681 | 17 | -0.0769 | 0.0475 | 0.1049 | 9419 |
| avg_sao2.males | rs113515681 | 17 | 0.0127 | 0.0598 | 0.8311 | 9663 |
| min_sao2 | rs113515681 | 17 | 0.1822 | 0.1984 | 0.3586 | 18979 |
| min_sao2.females | rs113515681 | 17 | 0.3244 | 0.2788 | 0.2445 | 9419 |
| min_sao2.males | rs113515681 | 17 | 0.0066 | 0.2781 | 0.981 | 9658 |
| percent_sleep_under_90_saturation | rs113515681 | 17 | -0.3048 | 0.1386 | 0.02791 | 19092 |
| percent_sleep_under_90_saturation.females | rs113515681 | 17 | -0.0878 | 0.1413 | 0.5345 | 9462 |
| percent_sleep_under_90_saturation_males | rs113515681 | 17 | -0.3166 | 0.2464 | 0.1988 | 9728 |
| rdi_3p | rs113515681 | 17 | -0.7828 | 0.4283 | 0.0676 | 19073 |
| rdi_3p_females | rs113515681 | 17 | -0.4035 | 0.4965 | 0.4165 | 9446 |
| rdi_3p_males | rs113515681 | 17 | -0.6619 | 0.6741 | 0.3262 | 9725 |
| avg_desat | rs13087058 | 3 | 0.0328 | 0.0227 | 0.149 | 10060 |
| avg_desat_females | rs13087058 | 3 | 0.0291 | 0.0298 | 0.3297 | 4117 |
| avg_desat_males | rs13087058 | 3 | 0.0226 | 0.0308 | 0.4617 | 5943 |
| avg_sao2 | rs13087058 | 3 | -0.0116 | 0.0131 | 0.3768 | 21127 |
| avg_sao2_females | rs13087058 | 3 | -0.0043 | 0.0156 | 0.7823 | 10647 |
| avg_sao2.males | rs13087058 | 3 | -0.0242 | 0.0208 | 0.2441 | 10480 |
| min_sao2 | rs13087058 | 3 | -0.0386 | 0.0652 | 0.5539 | 21128 |
| min_sao2.females | rs13087058 | 3 | -0.0363 | 0.0895 | 0.6846 | 10652 |
| min_sao2.males | rs13087058 | 3 | -0.0252 | 0.0926 | 0.7857 | 10476 |
| percent_sleep_under_90_saturation | rs13087058 | 3 | 0.0236 | 0.0477 | 0.6208 | 21260 |
| percent_sleep_under_90_saturation.females | rs13087058 | 3 | 0.0544 | 0.0467 | 0.2444 | 10714 |
| percent_sleep_under_90_saturation_males | rs13087058 | 3 | -0.0212 | 0.087 | 0.8073 | 10546 |
| rdi_3p | rs13087058 | 3 | 0.0639 | 0.1425 | 0.654 | 21244 |
| rdi_3p_females | rs13087058 | 3 | 0.1606 | 0.1616 | 0.3203 | 10697 |
| rdi_3p_males | rs13087058 | 3 | -0.0124 | 0.2268 | 0.9564 | 10547 |
| avg_desat | rs227724 | 17 | -0.0276 | 0.0224 | 0.2179 | 10060 |
| avg_desat_females | rs227724 | 17 | -0.0065 | 0.0283 | 0.8175 | 4117 |
| avg_desat_males | rs227724 | 17 | -0.0419 | 0.0311 | 0.1782 | 5943 |
| avg_sao2 | rs227724 | 17 | -0.0138 | 0.0132 | 0.295 | 21127 |
| avg_sao2_females | rs227724 | 17 | -0.0154 | 0.0158 | 0.3288 | 10647 |
| avg_sao2.males | rs227724 | 17 | -0.0171 | 0.0209 | 0.415 | 10480 |
| min_sao2 | rs227724 | 17 | -0.0145 | 0.065 | 0.8232 | 21128 |
| min_sao2.females | rs227724 | 17 | -0.0237 | 0.0891 | 0.7905 | 10652 |
| min_sao2.males | rs227724 | 17 | -0.0314 | 0.0928 | 0.7349 | 10476 |
| percent_sleep_under_90_saturation | rs227724 | 17 | -0.0281 | 0.0479 | 0.5575 | 21260 |
| percent_sleep_under_90_saturation.females | rs227724 | 17 | 0.0395 | 0.0473 | 0.4036 | 10714 |
| percent_sleep_under_90_saturation_males | rs227724 | 17 | -0.0742 | 0.0875 | 0.3963 | 10546 |
| rdi_3p | rs227724 | 17 | -0.0223 | 0.1425 | 0.8755 | 21244 |
| rdi_3p_females | rs227724 | 17 | 0.048 | 0.1621 | 0.7671 | 10697 |
| rdi_3p_males | rs227724 | 17 | -0.0997 | 0.2279 | 0.6617 | 10547 |

**Supplement Table 4** Summary statistics of our top NC or NCadjBMI SNPs evaluated in association with snoring. Summary statistics were downloaded from GWAS Central (<https://www.gwascentral.org>) and are based on GWAS published in Campos, García-Marín (19).

| **Chr** | **SNP** | **A1** | **A2** | **Freq** | **Beta** | **SE** | **P-value** |
| --- | --- | --- | --- | --- | --- | --- | --- |
| All people, BMI adjusted | | | | | | | |
| 17 | rs227724 | A | T | 0.65 | -0.005 | 0.001 | 6.5E-06 |
| 17 | rs113515681 | T | G | 0.97 | 0.005 | 0.003 | 0.13 |
| 3 | rs13087058 | T | C | 0.60 | 0.0006 | 0.001 | 0.58 |
| 12 | rs10771543 | G | A | 0.56 | 0.001 | 0.001 | 0.19 |
| 18 | rs62101076 | A | G | 0.98 | -0.003 | 0.003 | 0.38 |
| 7 | rs139177122 | A | G | 0.97 | -0.002 | 0.003 | 0.55 |
| All people, BMI unadjusted | | | | | | | |
| 17 | rs227724 | A | T | 0.66 | -0.005 | 0.001 | 1.6E-05 |
| 17 | rs113515681 | T | G | 0.97 | 0.0045 | 0.003 | 0.17 |
| 3 | rs13087058 | T | C | 0.60 | 0.001 | 0.001 | 0.36 |
| 12 | rs10771543 | G | A | 0.56 | 0.001 | 0.001 | 0.28 |
| 18 | rs62101076 | A | G | 0.98 | -0.004 | 0.003 | 0.26 |
| 7 | rs139177122 | A | G | 0.97 | -0.002 | 0.003 | 0.42 |
| Males only, BMI unadjusted | | | | | | | |
| 17 | rs227724 | A | T | 0.66 | -0.003 | 0.002 | 0.081 |
| 17 | rs113515681 | T | G | 0.97 | -1.1E-05 | 0.005 | 0.99 |
| 3 | rs13087058 | T | C | 0.60 | 0.001 | 0.002 | 0.52 |
| 12 | rs10771543 | G | A | 0.56 | -0.0002 | 0.002 | 0.9 |
| 18 | rs62101076 | A | G | 0.98 | 0.001 | 0.005 | 0.84 |
| 7 | rs139177122 | A | G | 0.97 | -0.002 | 0.005 | 0.62 |
| Females only, BMI unadjusted | | | | | | | |
| 17 | rs227724 | A | T | 0.66 | -0.006 | 0.001 | 1.5E-05 |
| 17 | rs113515681 | T | G | 0.97 | 0.008 | 0.004 | 0.051 |
| 3 | rs13087058 | T | C | 0.60 | 0.001 | 0.001 | 0.5 |
| 12 | rs10771543 | G | A | 0.56 | 0.002 | 0.001 | 0.14 |
| 18 | rs62101076 | A | G | 0.98 | -0.009 | 0.004 | 0.05 |
| 7 | rs139177122 | A | G | 0.97 | -0.003 | 0.004 | 0.5 |

**Supplement Table 5:** Co-localization analysis result for our top NC or NCadjBMI hits.

| **tissue** | **gene** | **Probability of colocalization** | **Most likely SNP** |
| --- | --- | --- | --- |
| Esophagus_Muscularis | NOG | 0.104 | rs227724 |
| Esophagus_Muscularis | PDZRN3 | 0.137 | rs13072073 |
| Esophagus_Gastroesophageal_Junction | NOG | 0.079 | rs227727 |
| Esophagus_Gastroesophageal_Junction | PDZRN3 | 0.096 | rs13087058 |
| Esophagus_Gastroesophageal_Junction | PDZRN3-AS1 | 0.000 | rs6766876 |
| Adipose_Subcutaneous | PDZRN3 | 0.000 | rs9818960 |
| Artery_Tibial | PDZRN3 | 0.238 | rs13087058 |
| Artery_Tibial | PDZRN3-AS1 | 0.108 | rs13087058 |
| Artery_Aorta | NOG | 0.000 | rs1286298 |
| Artery_Aorta | PDZRN3 | 0.148 | rs13087058 |
| Artery_Aorta | PDZRN3-AS1 | 0.153 | rs13087058 |
| Artery_Coronary | NOG | 0.000 | rs12437322 |
| Artery_Coronary | PDZRN3 | 0.093 | rs13072073 |
| Artery_Coronary | PDZRN3-AS1 | 0.001 | rs34210879 |
| Heart_Left_Ventricle | PDZRN3 | 0.090 | rs13087058 |
| Heart_Left_Ventricle | PDZRN3-AS1 | 0.017 | rs12637134 |
| Heart_Atrial_Appendage | NOG | 0.000 | rs12589636 |
| Heart_Atrial_Appendage | PDZRN3 | 0.077 | rs13087058 |
| Heart_Atrial_Appendage | PDZRN3-AS1 | 0.079 | rs13087058 |
| Colon_Sigmoid | PDZRN3 | 0.080 | rs13087058 |
| Muscle_Skeletal | FTO | 0.056 | rs11075993 |
| Esophagus_Gastroesophageal_Junction | FTO | 0.000 | rs12596457 |

**Supplement Table 6: Sample Characteristics of the Geisinger data set**

|  | **ALL** | **Females** | **Males** |
| --- | --- | --- | --- |
| **N** | 3297 | 1740 | 1557 |
| **Age (years)** | 55.09 (14.07) | 53.23 (13.81) | 57.16 (14.07) |
| **BMI kg/m2** | 37.24 (9.97) | 38.28 (10.83) | 36.09 (8.77) |
| **Height (m)** | 1.69 (0.17) | 1.62 (0.08) | 1.78 (0.21) |
| **Neck circumference (cm); Median/IQR** | 41.91 (38.10, 45.21) | 39.37 (36.32, 41.91) | 44.45 (41.91, 46.99) |
| **AHI (events/hr) median/IQR** | 6.20 (1.50, 17.40) | 4.20 (0.80, 11.40) | 9.50 (2.60, 25.30) |
| **Diabetes** | 1294, 39.25% | 638, 36.67% | 656, 42.13% |
| **History of CVD** | 678, 20.56% | 321, 18.45% | 357, 22.93% |
| **History of Atrial Fibrillation** | 397, 12.04% | 143, 8.22% | 254, 16.31% |
| **Insomnia** | 594, 18.02% | 385, 22.13% | 209, 13.42% |
| **Restless Legs syn/PLMD** | 290, 8.80% | 190, 10.92% | 100, 6.42% |
| **Narcolepsy** | 24, 0.73% | 20, 1.15% | 4, 0.26% |

**Supplement Table 7: Replication Results for the Top NCadjBMI Associations in Table 2 in the main text**

|  |  |  |  | **Sex-combined** | | | **Men** | | | **Women** | | |
| --- | --- | --- | --- | --- | --- | --- | --- | --- | --- | --- | --- | --- |
| **SNP** | **Chr** | **Gene** | **Alleles** | **N** | **Effect** | **P** | **N** | **Effect** | **P** | **N** | **Effect** | **P** |
| rs227724 | 17 | NOG | A/T | 3296 | -0.09 | 0.045 | 1554 | -0.121 | 0.045 | 1740 | -0.06 | 0.222 |
| rs113515681 | 17 | LOC105371884 | T/G | 3269 | -0.07 | 0.344 | 1544 | -0.168 | 0.235 | 1723 | 0.005 | 0.507 |
| rs13087058 | 3 | PDZRN3 | T/C | 3205 | -0.088 | 0.049 | 1511 | -0.084 | 0.128 | 1692 | -0.094 | 0.108 |
| rs10771543 | 12 | OVCH1 | A/G | 3108 | -0.056 | 0.145 | 1463 | 0.025 | 0.633 | 1643 | -0.123 | 0.053 |
| rs62101076 | 18 | LOC101927404 | A/G | 3272 | 0.289 | 0.550 | 1537 | 0.489 | 0.025 | 1733 | 0.159 | 0.275 |
| rs139177122 | 7 | RN7SKP75 | A/G | 3257 | -0.27 | 0.590 | 1537 | -0.423 | 0.041 | 1720 | -0.176 | 0.235 |

# Supplemental Figures

**Supplement Figure 1:** The eQTL result for the SNP rs227724 with the NOG gene.


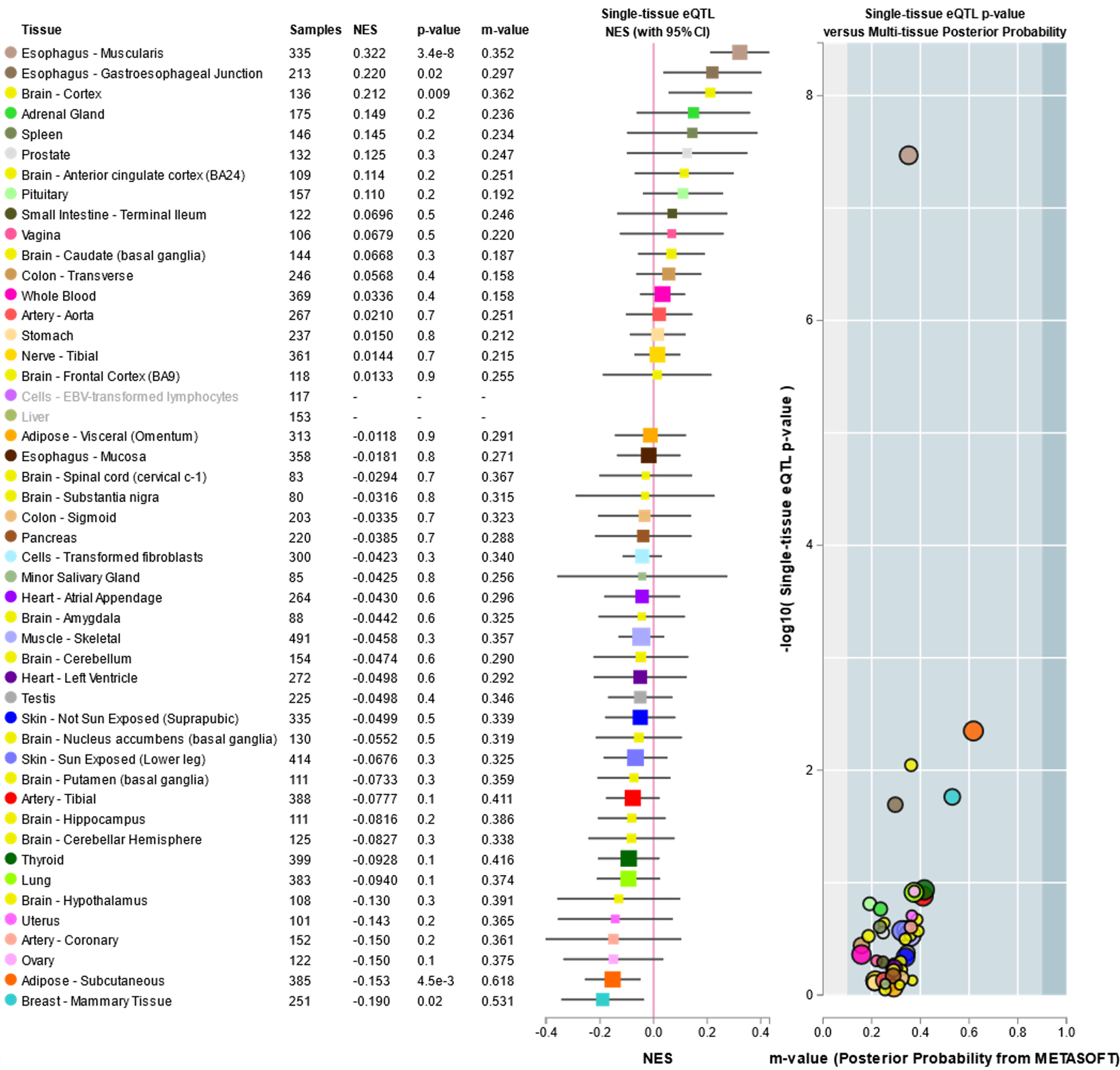


**Supplement Figure 2:** The eQTL result for the SNP rs13087058 with the PDZRN3 gene.





**Supplement Figure 3:** The eQTL result for the SNP rs56094641 with the FTO gene.


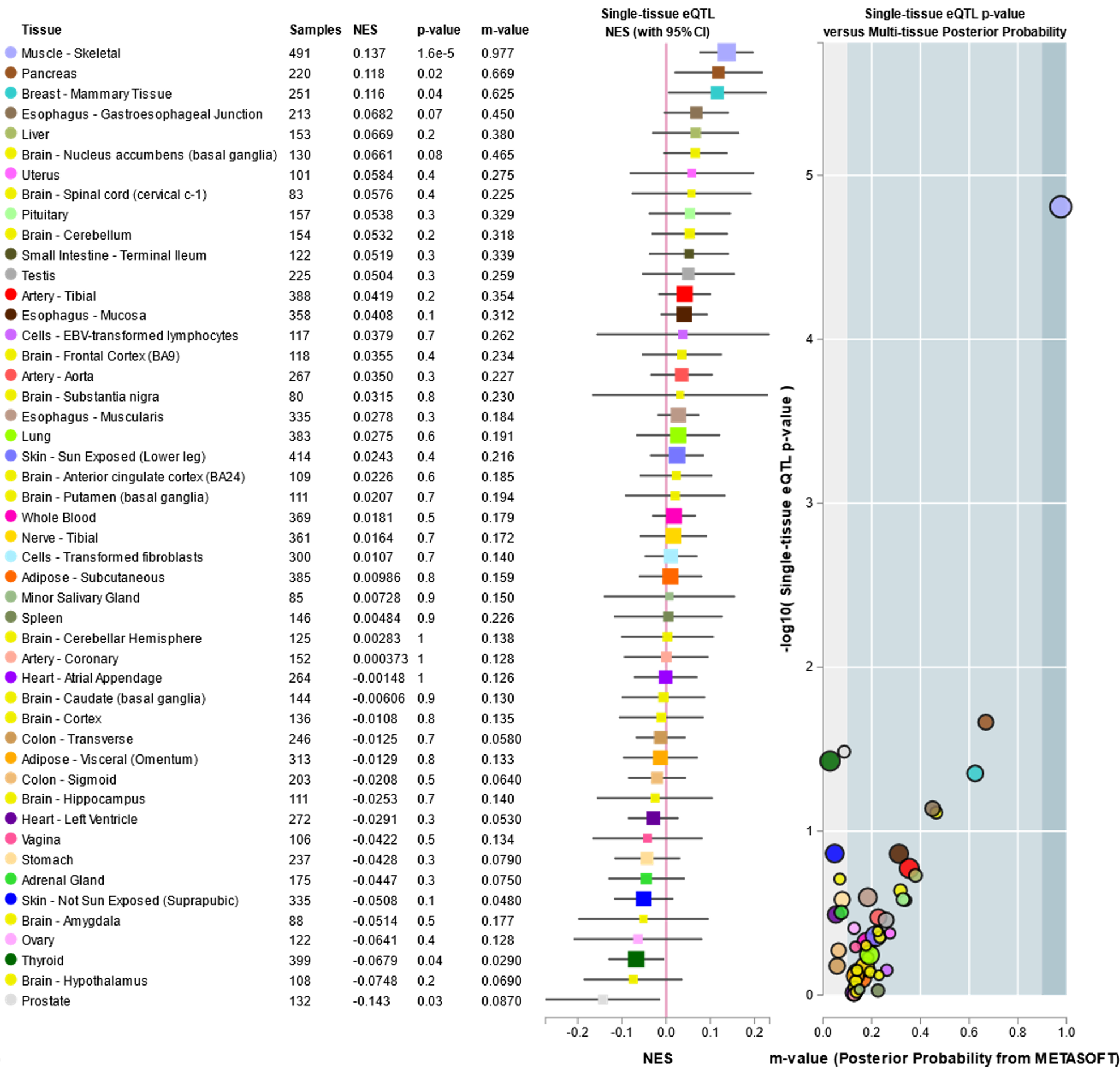

Supplement: Supplementary file 1 — Supplementary Materials [file 41366_2021_817_MOESM1_ESM.docx]
